# Detection of Kita-Kyushu Lung Cancer Antigen-1, a Cancer/Testis Antigen, in the Stomach Close to a Cancerous Condition

**DOI:** 10.7150/jca.67534

**Published:** 2022-10-31

**Authors:** Toshikazu Otsuka, Takashi Fukuyama, Nobue Futawatari, Kumiko Tahara, Masaaki Watanabe, Yoshinobu Ichiki, Takafumi Soeno, Yoshihito Takahashi, Hitoshi Yamazaki, Yuma Fujimori, Taichi Ohshiro, Noritada Kobayashi, Mitsuhiro Kida, Wasaburo Koizumi, Chika Kusano

**Affiliations:** 1 Division of Internal Medicine, Kitasato University Medical Centre, Kitamoto, Saitama, Japan.; 2 Division of Biomedical Research, Kitasato University Medical Centre, Kitamoto, Saitama, Japan.; 3 Division of General and Gastroenterological Surgery, Department of Surgery (Ohashi), Toho University, Tokyo, Japan.; 4 Department of General Thoracic Surgery, National Hospital Organization, Saitama Hospital, Wako, Saitama, Japan.; 5 Division of Surgery, Kitasato University Medical Centre, Kitamoto, Saitama, Japan.; 6 Division of Pathology, Kitasato University Medical Centre, Kitamoto, Saitama, Japan.; 7 Department of Microbial Chemistry and Medicinal Research Laboratories, Graduate School of Pharmaceutical Sciences, Kitasato University, Tokyo, Japan.; 8 Department of Gastroenterology, School of Medicine, Kitasato University, Sagamihara, Japan.

**Keywords:** gastric cancer, *Helicobacter pylori*, atrophic gastritis, Kita-Kyushu lung cancer antigen-1

## Abstract

**Background:** Kita-Kyushu lung cancer antigen-1 (KK-LC-1), encoded by *CT83*, is a cancer/testis antigen (CTA) and an attractive target for immunotherapy. Our previous study demonstrated frequent *CT83* expression in gastric cancers (GCs) and non-tumor sites of the stomach with tumors. Additionally, there was a correlation with *Helicobacter pylori* (*Hp*) infection. Since it currently remains unclear whether KK-LC-1 is expressed in the stomach without GC, this study investigated KK-LC-1 expression in non-GC stomach.

**Methods:** We investigated differences in *CT83* gene expression at non-tumor sites of stomachs with or without tumors in 118 GC patients and 115 non-GC patients. Fisher's exact test was used for statistical analyses.

**Results:** *CT83* expression was detected in 77% of non-tumor sites in stomachs with tumors, which was significantly higher than in stomachs without tumors (7%, p < 0.0001). All patients with *CT83* expression at non-tumor sites of their stomachs without tumors carried *Hp*.

**Conclusion:*** CT83* appears to be rarely expressed in the atrophic stomach, and furthermore, a part of patients positive for its expression will develop GC in the future, suggesting that *CT83* expression is a useful marker for predicting GC.

## Introduction

Gastric cancer (GC) is the third leading cause of cancer-related death worldwide 1. In Japan, the incidence rate of GC is the second highest of all cancers. Most cases are caused by *Helicobacter pylori* (*Hp*) infection 2. Therefore, detection and eradication of* Hp* infection may reduce the risk of GC. Furthermore, GC risk can be diagnosed by ABC classification using an anti-*Hp* antibody and measurement of pepsinogen I/II serum levels 3. However, this approach has proven to be inaccurate and indicates the occurrence of only 1%-5% of GC in high-risk groups. Thus, more precise methods are needed for risk diagnosis 4.

To date, numerous tumor-associated antigens have been identified in various human cancers 5. Among them, cancer/testis antigens (CTAs) are particularly attractive targets for immunotherapy. Such antigens have minimal or no expression in normal tissues, except for germline tissues, but they are aberrantly expressed in a range of human cancers 6. CTAs may be advantageous targets for systemic cancer diagnosis because of their specific and broad expression patterns in various cancer types. However, it has been shown that the individual CTA expression rate is insufficient for diagnostic applications 7.

Among known CTAs, Kita-Kyushu lung cancer antigen-1 (KK-LC-1), encoded by *CT83*, includes epitope peptides recognized by cytotoxic T lymphocytes (CTLs). CTLs against KK-LC-1 predominantly accumulate among tumor-infiltrating lymphocytes, which leads to a good response to adaptive immunotherapy 8. Except for the testis, KK-LC-1 is not expressed in normal tissues. It is expressed in 33%, 82%, and 75% of non-small cell lung cancers, GC, and triple-negative breast cancers, respectively 9-12.

In a previous study, we reported that* Hp* infection induces the expression of specific CTAs in addition to causing malignant transformation of host cells. Additionally, we found a correlation between *CT83* expression and *Hp* infection in clinical GC 13, 14. These findings suggest a correlation between KK-LC-1 expression and *Hp* infection, an early cancer-causing event. Therefore, KK-LC-1 may be a novel candidate for cancer prediction and diagnosis.

The expression rate of KK-LC-1 is 79% during early stages of GC 15, which indicates that KK-LC-1 expression occurs at the beginning of a malignancy and is subsequently maintained. Furthermore, we have observed that KK-LC-1 is expressed in premalignant lesions of the stomach 16. However, to date, it remains to be clarified whether the non-premalignant stomach expresses KK-LC-1. In the present study, we investigated the difference in *CT83* expression at non-tumor sites of stomachs with or without tumors. Additionally, we assessed the clinical utility of *CT83* for surveillance of GC occurrence.

## Materials and methods

### Patients

Patients underwent an endoscopic survey of the esophagus, stomach, and duodenum. In cases of an atrophic stomach, two portions, one from the lower and one from the middle corpus of the stomach, were sampled and subjected to a rapid urease test (RUT). After RUT confirmation, we collected gastric specimens. Between March 2016 and August 2017, 412 patients underwent RUTs at Kitasato University Medical Centre. Of these patients, a subset (n = 199) provided informed consent after RUT. Each specimen was checked for RNA quality by expression of β-actin (*ATCB*), and specimens with a threshold cycle (Ct) greater than 30 were excluded. Finally, 115 samples were used in the present study.

Between August 2012 and June 2017, 196 patients underwent surgical resection for GC at the Department of Surgery, Kitasato University Medical Centre, Kitamoto, Japan. We obtained tumor samples from stomachs with GC, as well as two or four non-tumor samples collected from random locations far from the tumor site and each non-tumor site. On the basis of *ACTB* expression levels, the patients whose one or more specimens were greater than 30 of *ACTB* expression were excluded in this study. all samples had sufficient mRNA quality. Finally, we enrolled 118 GC patients in the present study whose clinicopathological characters are shown in Supplementary Table S1. Furthermore, to compare RUT specimens, we selected 22 GC patients with two non-tumor samples from the lower and middle corpuses. The clinicopathological findings were classified in accordance with the Japanese Classification of Gastric Carcinoma (14^th^ edition) 17.

The Human Ethics Review Committee of Kitasato University Medical Centre, Japan (approval nos. 29-16 and 29-18) approved the study protocol. All experiments were performed in accordance with the relevant guidelines and regulations, and all patients signed informed consent prior to resection of the tissue samples used in this study.

### Tissue specimens

After collection, each specimen was immediately stored at 4°C overnight in RNAlater (Life Technologies, Carlsbad, CA, USA). Samples were subsequently stored at -80°C until use. For samples from GC patients, we performed hematoxylin-eosin staining on samples from each adjacent site to confirm the predominance of tumor cells at tumor sites and to establish that there was no contamination of tumor cells at non-tumor sites. Furthermore, we categorized the samples from non-tumor sites into fundic gland, borderline (B), or pyloric gland (P) samples.

### Expression analysis of CTAs

**Figure 1:**
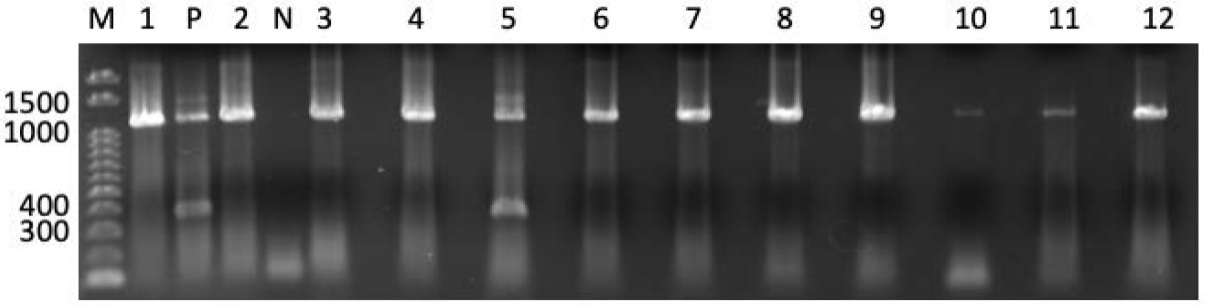
** Representative panel of *CT83* expression in noncancerous gastric specimens.** *CT83* expression was detected as an amplicon band of 342 bp. *CT83* expression was detected in 1 of 12 stomach samples (lane No. 5). Bands between 1000 and 1500 indicate amplicons of genomic DNA of *CT83*. M, 100 bp ladder; P, positive control (cDNA of MKN45 cell line); N, negative control.

We used the QIACUBE with the RNeasy Tissue Mini Kit (Qiagen, Hilden, Germany) to isolate total RNA from each sample in accordance with the manufacturer's instructions. Total RNA was converted to cDNA using oligo p(dN)_6_ random primers and Superscript III reverse transcriptase (Life Technologies). *ACTB*, melanoma antigen gene (*MAGE*) A1, *MAGEA3*, *MAGEA4*, New York esophageal carcinoma-1 (*CTAG1A*), and synovial sarcoma, X breakpoint-4 (*SSX4*) expression was measured with TaqMan Gene Expression Assays (IDs: Hs99999903_m1, Hs00607097_m1, H200366532_m1, Hs00365979_m1, Hs00265824_m1, and Hs02341532_m1, respectively). A 7900HT Fast Real-Time PCR System (Life Technologies) was used to perform the analyses. The threshold cycle number of cDNAs converted from RNAs was measured for *ACTB*. Real-time polymerase chain reaction (PCR) was performed in a 20 µL volume consisting of 5 µL cDNA template, 10 µL FastStart Universal Probe Master Mix (Roche, Mannheim, Germany), and 1 µL TaqMan Gene Expression Assay. Samples were qualified by ≤30 threshold cycles (C_T_) of *ACTB* and then assessed for CTA expression. The expression of CTAs except for *CT83* was assessed as positive with ≤45 C_T_. *CT83* expression was examined by 40-cycle endpoint reverse transcription (RT)-PCR because an appropriate probe for *CT83* mRNA detection was unavailable. PCR amplification was performed in a 20 µL volume consisting of 2 µL cDNA template, rTaq (Takara, Tsu, Japan), dNTPs (Roche, Basel, Switzerland), and 500 nM each of gene-specific primers ATGAACTTCTATTTACTCCTAGCGAGC and TTAGGTGGATTTCCGGTGAGG (Sigma-Aldrich Japan, Tokyo, Japan). The annealing temperature was 67°C. We performed 40 cycles to yield a 342-bp product. PCR products were visualized by ethidium bromide staining and ultraviolet light exposure, following electrophoresis on a 1.5% agarose gel. A representative panel for detection of *CT83* expression in tumor-free atrophic stomachs is shown in Figure 1.

### Statistical analysis

Fisher's exact test was used for the comparison between *CT83* expression and each clinicopathological factor. JMP14.0 (SAS Institute Japan, Tokyo, Japan) was used for the analysis.

## Results

### CTA expression in GC tumors

We used RT-PCR analysis to determine the fractions of the tumor sites expressing specific CTA genes. Of the 118 GC tumors evaluated, 92 specimens (78.0%) expressed* CT83* (Table 1). We found that other CTAs, specifically *MAGEA1*, *MAGEA3*, *MAGEA4*, *CTAG1A*, and *SSX4* had expression frequencies of 29.7%, 32.2%, 19.5%, 13.6%, and 17.8%, respectively.

### CT83 expression in non-tumor sites of stomachs with or without tumors

In 69 out of 118 (58.5%) GC patients, *CT83* was detected at one or more non-tumor sites (Table 2). There was no significant difference in the positive rate between GC patients with two and four sampled sites (p = 0.8517, Supplementary Table S2).

We investigated GC patients in which samples were obtained from the lower and middle corpuses of the same two non-tumor sites of non-GC patients. In 17 of 22 (77.3%) patients, *CT83* was expressed at either of the two or both of the two non-tumor sites at significantly higher rates than in the remaining GC patients (45.7%, p = 0.0276, Supplementary Table S3).

Conversely, among non-GC patients with gastric atrophy, we identified 8 of 115 (7.0%) with *CT83* expression (Table 2). Of note, the* CT83* expression rate in non-GC patients was significantly lower than that in GC patients (p < 0.0001, Table 3). There were no significant differences in the detection of *CT83* expression among age, gender, and atrophic severity. All *CT83*-positive patients were infected with *Hp* (Table 3). When exclusively considering *Hp*-positive non-GC patients, we observed 11.6% with *CT83* expression (p < 0.0001, Table 3). Supplementary Table S4 lists non-GC patients with *CT83* expression. Except for patient NGC#006, who had a mass in the stomach at the time of endoscopic survey, no specific clinical features except *Hp* infection were observed in patients with positive *CT83* expression; for NGC#006, adenoma was diagnosed on biopsy of a mass in a different site from the biopsy analyzed for *CT83* expression. After 2 months of *CT83* analysis, patient NGC#006 underwent endoscopic submucosal dissection to remove the adenoma. The final pathological diagnosis was adenocarcinoma in adenoma. Furthermore, the patient relapsed with gastric cancer after 14 months of *CT83* analysis, despite successful treatment for *Hp* eradication. In addition, gastric cancer occurred in patient #003 after 49 months of *CT83* expression analysis, even though the patient had received successful treatment for *Hp* eradication.

## Discussion

**Figure 2:**
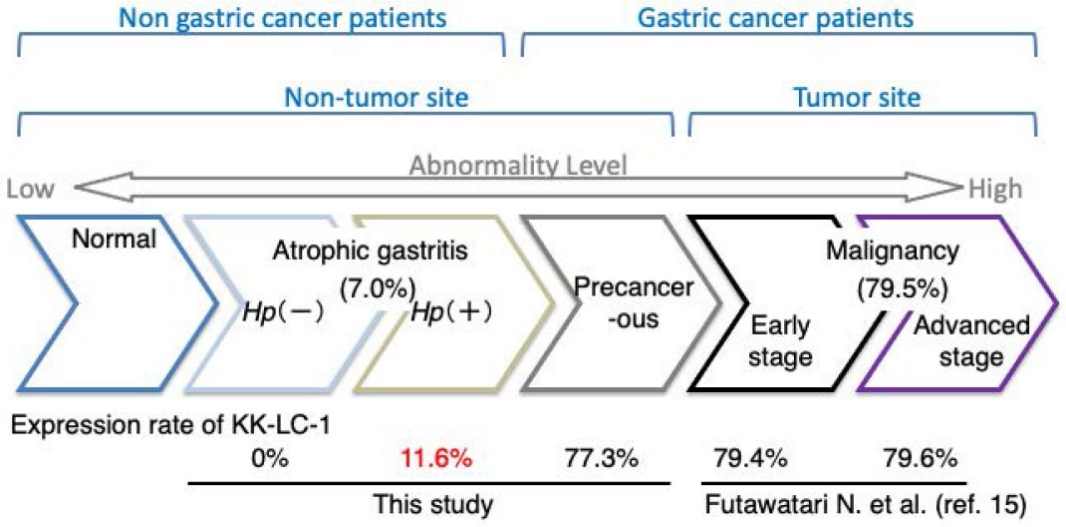
** Expression rate of *CT83* during the transition to gastric abnormality.** The expression rate of *CT83* is summarized for each stage of gastric abnormality. *CT83* was not expressed in the stomach under normal conditions. However, with the development and progression of gastric abnormality, *CT83* expression was increased in the following order: atrophic gastritis without and with Hp infection, non-tumor sites of the precancerous stomach with gastric cancer, and early and advanced gastric cancer tumors. This study showed that *CT83* expression was clearly discrepant between non-cancerous and cancerous stomachs regardless of *Hp* infection. The *CT83* expression rate in malignancy including early and progressive stages was adopted from Futawatari *et al.* 15. *Hp*, *Helicobacter pylori*.

KK-LC-1 is classified as a CTA because of its absence in normal tissues except for the testis. However, it is expressed in cancers of multiple organs 7, 10. Furthermore, Fukuyama *et al.* found a correlation between KK-LC-1 expression and *Hp* infection. Additionally, we previously showed that *CT83* and KK-LC-1 expression was frequently detected at non-tumor sites of the stomach with GC 14, 16. However, it remained unclear whether KK-LC-1 is expressed in the stomachs of patients with* Hp* infection and without tumors. In the present study, KK-LC-1, encoded by *CT83*, was frequently expressed in non-tumor sites of the GC stomach, but infrequently in those of the non-GC stomach (Fig. 2). Furthermore, while* CT83* expression was correlated with* Hp* infection, it was not an exclusive indicator of *Hp* infection. There is also a similar report by Watari et al. showing greater methylation changes in miR-124a-3 in the order of Hp+/GC, Hp+/AG, and Hp-/AG 18. The difference between their study and ours is that our subject is detected with higher frequency in GC patients and less frequently in AG patients. Furthermore, we also confirm that carcinogenesis occurs in the object-positive group but not in the object-negative group, although further case accumulation is needed. *Hp* infection, ABCD stratification, and atrophic severity, which are currently considered predictors of carcinogenesis, have been associated with higher cumulative incidence of gastric cancer in high-risk groups 2, 19, 20. Other new and potential predictive markers of carcinogenesis, including *CT83* expression, also need to be followed up to determine whether their positive cases will show carcinogenesis in the future 21.

*CT83* expression might be a new indicator to measure precancerous levels in the stomach. Higher* CT83* expression in non-tumor sites with GC compared with non-GC stomachs may indicate precancerous levels. Of note, epigenetic alterations are precancerous level indicators. Compare *et al.* reported that global hypomethylation of the gastric mucosa gradually advances from *Hp*-negative gastritis to *Hp*-positive gastritis, *Hp*-positive chronic gastritis, and gastric carcinoma 22. Conversely, Leodolter *et al.* showed that global hypomethylation occurs during the early stages of gastritis regardless of *Hp* infection 23. Detection of *CT83* expression may facilitate determining whether the stomach is close to a malignant level independent of the accumulation of genetic/epigenetic alterations.

*CT83* expression was not detected at non-tumor sites of the stomachs in three GC patients. However, its expression was detected in tumor sites of the same patients. Such an issue may be resolved by sampling multiple mucosa sites from the lower corpus. Supplementary Figure S1 and our previous study showed that *CT83* expression rates in the lower corpus were 68.1% and 66.1%, respectively 16.

We observed two non-GC patients whose non-tumor sites of the stomach expressed *CT83*. These patients were later diagnosed with GC. Of note, the area of each specimen of the two patients differed from the area in which the tumor later occurred. This phenomenon suggests that *CT83* expression indicate the presence of GC in stomach allopatric sites.

Additionally, *CT83* expression was observed in non-tumor sites of GC patients. Of note, GC patients who underwent mucosal resection, including ESD, more frequently presented with metachronous cancers from gastric remnants than during surgical resection 24. These findings suggest that *CT83* expression is a predictive marker of GC occurrence. Non-GC patients in whom *CT83* expression was detected are under follow-up to identify possible GC development. However, because all non-GC patients with *CT83* had received antibiotics against *Hp*, genetic/epigenetic alterations caused by *Hp* will not accumulate, which in return will prevent GC from developing. Nonetheless, GC can occur following *Hp* eradication 25. The occurrence of GCs in non-GC patients in whom *CT83* expression was detected indicates the potential of KK-LC-1 as a new predictive marker for GC.

## Conclusion

*CT83* was frequently expressed in non-tumor sites in the GC stomach, but rarely in the non-GC stomach. Furthermore, all non-GC patients with *CT83* expression had *Hp* infection, and part of these patients would develop GC in the future. These results indicate that *CT83* is rarely expressed in the atrophic state, but is expressed in those particularly close to the precancerous state, in which carcinogenesis is of high potential. The findings in this study indicate the potential of *CT83* as a useful marker for GC prediction.

## Supplementary Material

Supplementary figure and tables.

## Figures and Tables

**Table 1:** Cancer/testis antigen (CTA) expression in 118 gastric cancer tumors

CTA	Positive	Negative	Frequency (%)
*CT83*	92	26	78.0
*MAGEA1*	35	83	29.7
*MAGEA3*	38	80	32.2
*MAGEA4*	23	95	19.5
*CTAG1A*	16	102	13.6
*SSX4*	21	97	17.8

*CT83*, Kita-Kyushu lung cancer antigen-1; *MAGE*, melanoma antigen gene; CTAG1A, New York esophageal squamous cell carcinoma-1; *SSX4*, synovial sarcoma, X breakpoint-4.

**Table 2:** *CT83* expression with patient characters of atrophic gastritis

Clinicopathological parameters	Categories	Total	*CT83* expression	
		(n=115)	Positive	Negative
Age (average ± SD)	-	66.1 ± 12.0	67.4 ± 8.6	66.1 ± 12.2
Gender	Male	64 (56%)	5 (8%)	59 (92%)
	Female	51 (44%)	3 (6%)	48 (94%)
Hp infection	Positive	69 (60%)	8 (12%)	61 (88%)
	Negative	46(40%)	0 (0%)	46 (100%)
Atrophic severity	High (O1-O3)	65 (57%)	6 (9%)	59 (91%)
	Low (C1-C3)	36 (31%)	2 (6%)	34 (94%)
	Not recorded	14 (12%)	0 (0%)	14 (100%)
*CT83* expression	Positive	8 (7%)	-	-
	Negative	107 (93%)	-	-

Values are presented as number (%) or mean ± standard deviation. *CT83*; Kita-kyushu lung cancer antigen-1, Hp; *Helicobacter pylori*. Atrophic severity was classified as high and low with O1 to O3 and C1 to C3 of Kimura-Takemoto classification, respectively.

**Table 3:**
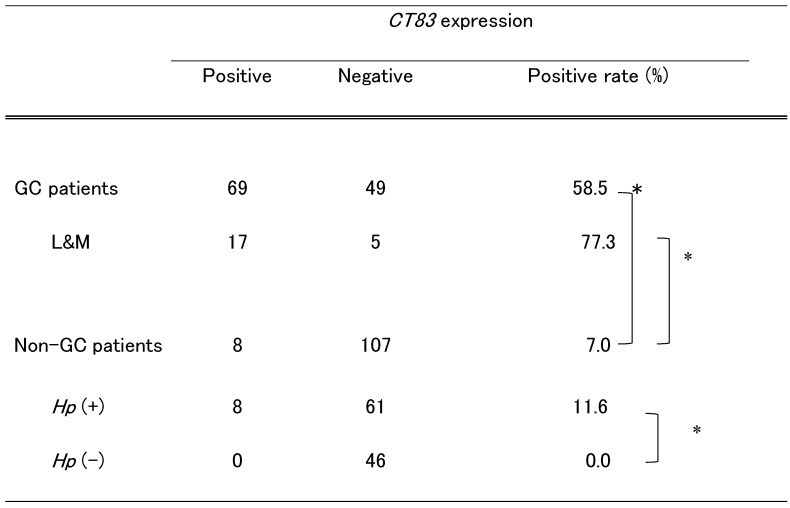
*CT83* expression at non-tumor sites of GC and non-GC patients

*CT83*, Kita-Kyushu lung cancer antigen-1; GC, gastric cancer; L&M, each lower and middle corpus sampled; *Hp*, *Helicobacter pylori*. *p < 0.0001.

## Abbreviations

GC: gastric cancer
*Hp*: *Helicobacter pylori*
CTA: cancer/testis antigen
KK-LC-1: Kita-Kyushu lung cancer antigen-1
CTL: cytotoxic T lymphocyte
RUT: rapid urease test
RT-PCR: reverse transcription-polymerase chain reaction
MAGE: melanoma antigen gene
B: borderline
P: pyloric gland
ESD: endoscopic submucosal dissection
