# Supplementary material for: Detection of Kita-Kyushu Lung Cancer Antigen-1, a Cancer/Testis Antigen, in the Stomach Close to a Cancerous Condition
Source: J Cancer. 2022 Oct 31;13(14):3526–32. doi: 10.7150/jca.67534 (PMC9723985; doi:10.7150/jca.67534)
Supplement: Supplementary file 1 — Supplementary figure and tables. [file jcav13p3526s1.pdf]

Supplementary Table S1. Patient characters of gastric cancer

**Clinicopathological parameters**	**Categories**	**n=118**
Age	-	70.4 ± 10.0
Gender	male	69 (58.5%)
	female	49 (41.5%)
Laparoscopic approach	-	41 (34.7%)
Operatation procedures	Proximal gastrectomy (PGR)	2 (1.7%)
	Distal gastrectomy (DGR)	77 (65.3%)
	Pyrolus preserving gastrectomy(PPG)	4 (3.4%)
	Total gastrectomy (TGR)	35 (29.7%)
Lymph node dissection	D0	1 (0.9%)
	D1	15 (12.7%)
	D1+	47 (39.8%)
	D2	55 (46.6%)
ASA physical status classification	I	30 (25.4%)
	II	79 (66.1%)
	III	10 (8.5%)
Location	Upper	22 (18.6%)
	Middle	43 (36.4%)
	Lower	53 (44.9%)
Tumor size (mm)	-	50.8 ± 30.0
Multicentric cancer	-	5 (4.2%)
Macroscopic type (pathological)	0	45 (38.1%)
	1	4 (3.4%)

	2	35 (29.7%)
	3	21 (17.8%)
	4	9 (7.6%)
	5	4 (3.4%)
Lymphatic permeation	ly0	34 (28.8%)
	ly1	46 (39.0%)
	ly2	23 (19.5%)
	ly3	15 (12.7%)
Vascular permeation	v0	45 (38.1%)
	v1	32 (27.1%)
	v2	27 (22.9%)
	v3	14 (11.9%)
Postoperative adjuvant therapy	-	47 (39.8%)
JGCA 14th T factor	pT1a	14 (11.9%)
	pT1b	36 (30.5%)
	pT2	16 (13.6%)
	pT3	23 (19.5%)
	pT4a, pT4b	29 (24.6%)
JGCA 14th N factor	pN0	59 (50.0%)
	pN1	21 (17.8%)
	pN2	14 (11.9%)
	pN3	24 (20.3%)
JGCA 14th pStage	Ia	37 (31.4%)
	Ib	15 (12.7%)
	IIa	17 (14.4%)
	IIb	13 (11.0%)
	IIIa	3 (2.5%)
	IIIb	9 (7.6%)
	IIIc	7 (5.9%)
	IV	17 (14.4%)

Values are presented as mean ± standard deviation or number (%).

ASA; American Society of Anesthesiologists , JGCA; Japanese Classification of Gastric Cancer.

Supplementary Table S2

Number of GC patients with *CT83* expression at non-tumor sites

GC patients	Total	Positive	Negative	Positive rate (%)	
Total	118	69	49	58.5	
Two sites sampled	57	32	25	56.1	p=0.8517
Hp (+)	47	28	19	59.6	p=0.7838
Hp (−)	10	4	6	40.0	
Four sites sampling	61	37	24	60.7	
Hp (+)	44	28	16	63.6	p=0.5611
Hp (−)	17	9	8	52.9	

GC patients were divided into two groups with two and four regions of non-tumor sites sampled each. CT83, Kita-Kyushu lung cancer antigen-1; GC, gastric cancer

Supplementary Table
S3

CT83 expression of non-tumorous stomach among GC patients sampled at two regions

Sampling sites	*CT83* expression			
	Positive	Negative	Positive rate (%)	
L and M each	17	5	77.3	p = 0.0276
Others	16	19	45.7	

CT83, Kita-kyushu lung cancer antigen-1; GC, gastric cancer; L, lower corpus; M, middle corpus.

Supplementary Table S4

Non-GC patients with gastric atrophy and *CT83* expression

Non-GC No.	Age	Gender	Hp infection (RUT)	Kimura-Takemoto classification	Duration of GC occurrence after CT83 measure (month)	Remarks
001	51	M	Positive	C-1	-	Intestinal metaplasia
002	63	F	Positive	O-1	-	
003	69	M	Positive	O-3	49	Intestinal metaplasia
004	78	M	Positive	O-2	-	
005	70	F	Positive	O-3	-	Increased vascular permeability
006	62	M	Positive	O-2	2 (primary), 14 (secondary)	Adenoma of biopsy
007	76	M	Positive	C-3	-	
008	70	F	Positive	O-2	-	

GC, gastric cancer; CT83, Kita-Kyushu lung cancer antigen-1; Hp, *Helicobacter pylori*; RUT, rapid urease test

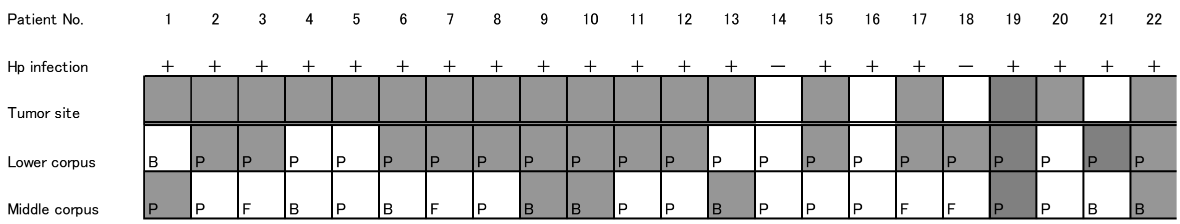

Supplementary Figure S1 *CT83* expression at tumour and non-tumour sites of each patient with gastric cancer (GC)

Each gastric specimen carrying a tumour was sampled as follows: one tumour site and two non-tumour sites (randomly and distant from the tumour). Patients with GC were selected using a study that sampled non-tumour sites from both the lower and middle corpus. *CT83* expression was evaluated in both the tumour and non-tumour sites of the specimen. Open or closed squares indicate *CT83*' s non-expression or expression, respectively. The adjacent sampled gland sites underwent hematoxylin and eosin staining and were distinguished as the pyloric gland (P), fundic gland (F) or their combination (borderline; B). When both ends were all P or F glands, the sampled site was considered P or F. When one end was a combination of P and F glands, the sampled site was considered B. If one end was P and the other was F, the sampled site was considered B. Hp: Helicobacter pylori; +: Hp infection positive; −: Hp infection negative.
